# Mastering your fellowship: Part 3 2026

**DOI:** 10.4102/safp.v68i1.6286

**Published:** 2026-06-08

**Authors:** Klaus B. von Pressentin, John M. Musonda, Gert Marincowitz, Selvandran Rangiah

**Affiliations:** 1 Division of Family Medicine, Department of Family, Community and Emergency Care (FaCE), Faculty of Health Sciences, University of Cape Town, Cape Town, South Africa; 2 Department of Family Medicine and Primary Care, Faculty of Health Sciences, The University of Witwatersrand, Johannesburg, South Africa; 3 Department of Family Medicine, Faculty of Health Sciences, University of Limpopo, Polokwane, South Africa; 4 Department of Family Medicine, College of Health Sciences, University of KwaZulu-Natal, Durban, South Africa

**Keywords:** family physicians, FCFP (SA) examination, anaesthesia, obstetric safety, clinical governance, evidence-based practice

## Abstract

The series ‘Mastering your Fellowship’ provides examples of the question formats encountered in the written and clinical examinations (Part A) of the FCFP (SA) examination. This edition is aligned to Entrustable Professional Activity (EPA) 6 – Providing anaesthesia in the district hospital operating theatre. Model answers are available online.

This section in the *South African Family Practice* journal aims to help registrars prepare for the FCFP (SA) Final Part A examination (Fellowship of the College of Family Physicians). It will provide examples of the question formats encountered in the written exam: Multiple Choice Question (MCQ) in the form of Single Best Answer (SBA – Type A) and Extended Matching Question (EMQ – Type R); Short Answer Question (SAQ), questions based on the Critical Reading of a Journal article (CRJ: evidence-based medicine) and an example of an Objectively Structured Clinical Examination (OSCE) question. Each of these question types is presented in accordance with the College of Family Physicians blueprint and the key learning outcomes of the FCFP (SA) programme. The MCQs draw on the 10 clinical domains of family medicine; the SAQs align with the five national unit standards; and the critical reading section covers evidence-based medicine and primary care research methods.

This edition is based on Entrustable Professional Activity (EPA) 6 – Providing anaesthesia in the district hospital operating theatre. We suggest that you attempt to answer the questions (either on your own or with peers and supervisors) before finding the model answers online at http://www.safpj.co.za/.

Please visit the Colleges of Medicine website for guidelines on the Fellowship examination: https://cmsa.co.za/fellowship-of-the-college-of-family-physicians-of-south-africa-fcfpsa/. We are keen to hear how this series helps registrars and their supervisors to prepare for the FCFP (SA) examination. Please email us your feedback and suggestions.

## Multiple choice question: Single Best Answer

A 28-year-old G2P1 woman presents to the district hospital for an emergency lower-segment caesarean section because of prolonged second stage of labour. After preparing the woman for surgery, a medical officer injects a single-shot hyperbaric, adrenaline-free bupivacaine 0.5% 1.8 mL (=9 mg) with 0.2 mL (=10 mg) fentanyl. After a while, the woman experiences discomfort and abdominal pain at the surgical site. She develops bilateral leg weakness and minimal cold sensation up to the knees, but pinprick sensation is intact at T10. Her BP = 103/62 mmHg (pre-spinal BP was 125/78), heart rate (HR) = 108/min, respiratory rate (RR) = 18 BPM, with clear lungs. As a family physician on call, you are contacted by the theatre team for an opinion. What is the most appropriate immediate next step for this woman?

- Administer the IV ketamine sedation and analgesia and proceed with the operation.
- Allow time to pass, assess the spinal effect and repeat it at a similar level, as needed.
- Convert to general anaesthesia, with rapid sequence induction and intubation.
- Repeat the spinal anaesthesia injection at a higher interspace than before.

### Short answer

c. Convert to general anaesthesia, with rapid sequence induction and intubation.

### Discussion

The National Committee on Confidential Enquiries into Maternal Deaths (NCCEMD) guidelines highlight that spinal anaesthesia is the first choice for caesarean sections. In addition, the use of spinal anaesthesia has risen because it is the preferred anaesthesia for caesarean sections, especially in low-resource settings, to improve foetal and maternal health outcomes. Therefore, it is essential to distinguish between a failed spinal block, in which no sensory or motor effects are experienced after administering the spinal anaesthesia, and a partial block, in which some motor and sensory effects are present but inadequate for surgery. Both situations cause the mother to experience pain because of the operation and an intervention is necessary.

Converting immediately to general anaesthesia is recommended, especially when clinicians are experienced in airway management and when the mother has a full stomach. To conduct a general anaesthesia safely, a skilled airway provider must be present to prepare the airway kit and resuscitation drugs properly and to ensure that assistance is immediately available. With a failed spinal, where no anaesthesia effect is experienced after some time has passed, repeating the spinal at a different interspace is not an acceptable salvage intervention, and it will not be helpful to wait any longer, as the operation is urgent.

Proceeding with the surgical operation under the current block and administering incremental IV ketamine and local infiltration is not advisable, although the intervention may provide dissociative anaesthesia. The risks of administering ketamine in this setting include loss of airway tone, inability to secure the airway and worsening haemodynamic instability. The woman has a motor block in the legs, but with a sensory level which is inadequate for the caesarean section, as indicated by a pinprick at T10. Augmenting the current partial block is not an acceptable option, where a general anaesthesia can safely be performed.

Re-attempting the spinal at a different interspace may be possible, where there are no signs of block, indicating a complete or technical failure because of the wrong drug or a failed intrathecal delivery, or where the operation is not an emergency. There is a risk of high or total spinal block.

### Further reading

- The ESMOE Anaesthesia Working Group. National Essential Steps in Managing Obstetric Emergencies (ESMOE). Guidelines for district and regional hospitals. Protocol for caesarean section under spinal anaesthesia [homepage on the Internet]. 2011 [cited 2025 Nov 08]. Available from: https://www2.kznhealth.gov.za/family/protocol_caesarean_section_under_spinal_anaesthesia.pdf
- The ESMOE Anaesthesia Working Group. Spinal anaesthesia – Important innervations and sensory dermatomes [homepage on the Internet]. [cited 2025 Nov 08]. Available from: https://www2.kznhealth.gov.za/family/Spinal_Anaesthesia_dermatomes.pdf
- South Africa Department of Health. National integrated maternal and perinatal care guidelines for South Africa [homepage on the Internet]. 5th ed. Pretoria: South Africa Department of Health; 2024 [cited 2025 Dec 19]. Available from: https://knowledgehub.health.gov.za/system/files/elibdownloads/2024-10/Integrated%20Maternal%20and%20Perinatal%20Care%20Guideline_23_10_2024_0.pdf.
- National Department of Health. National Committee for Confidential Enquiry into Maternal Deaths: Annual Report for 2023 [homepage on the Internet]. Pretoria: National Department of Health; 2024 [cited 2025 Dec 19]. Available from: https://www.health.gov.za/wp-content/uploads/2024/10/Saving-Mothers-Report-2023.pdf.

## Short Answer Question – Obstetric anaesthesia safety and clinical governance

You are the newly appointed family physician at a 250-bed district hospital with 24 medical officers. The facility performs approximately 400 deliveries and 100 caesarean sections monthly. Last month, there were two maternal deaths, both reported as anaesthesia-related. You chaired the 24-h mortality reviews. The District Manager requests an improvement strategy to prevent recurrence.

1. List at least eight key pieces of information you would extract from the maternity and anaesthesia records of both cases during the record audit. (8 marks)
2. Identify four additional areas of information or verification you need because these deaths were reported as anaesthesia-related. (4 marks)
3. Based on the two cases (high spinal with failed intubation; post-spinal hypotension progressing to postpartum haemorrhage and irreversible shock), propose three facility-level interventions to prevent recurrence (3 marks) and explain how you will implement each in a district hospital context (3 marks). (6 marks)
4. Outline seven key steps you will follow in managing these deaths as Patient Safety Incidents (PSIs) and explain how this supports clinical governance. (7 marks)

Total: 25 marks

### Suggested answers (the answers should show some application to the scenario)

1. **List at least eight key pieces of information you would extract from the maternity and anaesthesia records of both cases during the record audit. (8 marks)**
  *Note to examiner: Award up to 8 marks (≈1 mark per distinct relevant item). Credit breadth across antenatal, intra- or peri-operative, anaesthesia, monitoring, resuscitation, staffing and documentation*.
  - Antenatal profile and risk: booking gestational age, parity, comorbidities (hypertension, obesity, cardiac), risk scores or MEOWS (Modified Early Obstetric Warning Score); referral history.
  - Investigations and optimisation: Haemoglobin and platelets, HIV and antiretroviral therapy, abnormal results acted upon.
  - Indication and urgency of caesarean section (CS): decision-to-incision times; classification of urgency; intra-uterine resuscitation attempted.
  - Intrapartum monitoring: Cardiotocography (CTG) or foetal heart rate and maternal vital signs trends pre-theatre.
  - Pre-anaesthetic assessment: American Society of Anaesthesia (ASA) class; airway assessment (Mallampati, mouth opening, neck); fasting; allergies; previous anaesthesia issues.
  - Consent: procedure and anaesthesia consent; information provided and language used.
  - Anaesthetic record completeness: technique; drug name, dose and baricity; level and block height; patient position; fluids; vasopressor prophylaxis or treatment; complications.
  - Monitoring and equipment: SpO_2_, non-invasive blood pressure monitoring, Electrocardiogram (ECG)capnography for general anaesthesia; machine and suction checks; oxygen availability.
  - Airway events: sequence and number of attempts; adjuncts; use of supraglottic airway; front-of-neck access readiness.
  - Surgical and obstetric notes: quantified blood loss; uterotonics used; tranexamic acid (TXA) timing; haemostasis steps.
  - Fluids, blood and products: intravenous access size; volumes; massive transfusion protocol (MTP) activation; product ratios; calcium use.
  - Resuscitation chronology: timeline; roles; reversible causes; outcome.
  - Team composition and seniority: who anaesthetised; supervision; senior attendance after hours.
  - Post-event actions: debrief; family communication; immediate incident notification.
2. **Identify four additional areas of information or verification you need because these deaths were reported as anaesthesia-related. (4 marks)**
  *Note to examiner: one mark each; accept any four high-yield areas below or equivalents*.
  - Equipment readiness and maintenance: Daily machine check logs, difficult airway trolley, capnography, suction, oxygen backup, front-of-neck kit.
  - Drug availability and protocols: Vasopressors (phenylephrine, metaraminol or ephedrine), lipid emulsion for LAST (local anaesthetic systemic toxicity), uterotonic agents, TXA, dosing charts.
  - Staffing and competence: Skills mix, sign-off for spinal or general anaesthesia and caesarean section; senior cover pattern; induction and continuous professional development (CPD), including simulation records.
  - Safety systems: World Health Organization (WHO) Surgical Safety Checklist, obstetric anaesthesia checklist, escalation pathways, failed intubation algorithm, postpartum haemorrhage (PPH) bundle and MTP.
3. **Based on the two cases (high spinal with failed intubation; post-spinal hypotension progressing to postpartum haemorrhage and irreversible shock), propose three facility-level interventions to prevent recurrence (3 marks) and explain how you will implement each in a district hospital context (3 marks). (6 marks)**
  *Note to examiner: Award one mark for each appropriate intervention (max 3) and one mark for a feasible, context-appropriate implementation detail for each (max 3)*.

#### Standardise safe neuraxial anaesthesia for caesarean section

Intervention: protocol for dose and positioning, fluid co-load, prophylactic vasopressor, target block height, monitoring schedule, management of high or total spinal.

Implementation: one-page poster; briefing; integrate into WHO checklist; monthly mini-audit of 10 CS charts; ensure phenylephrine or metaraminol stock; update crash trolley.

#### Difficult airway readiness in obstetrics

Intervention: obstetric difficult airway cart (bougie, second-generation supraglottic, video laryngoscope if available, scalpel-bougie kit); failed intubation algorithm; capnography mandatory for general anaesthesia (GA).

Implementation: skills audit & sign-off for failed intubation and front-of-neck access; quarterly simulation; equipment checklist with named responsibility; early senior call rule.

#### Haemorrhage and resuscitation bundle integration

Intervention: PPH bundle (quantified blood loss, uterotonics sequence, TXA within 3 h, tamponade, rapid transfusion, calcium), MTP triggers and ratios, shock or MEOWS pathway.

Implementation: monthly obstetric emergency drills; ensure group O or crossmatch access; role badges during drills; laminate MTP; ensure TXA presence in theatre and recovery; plan-do-study-act (PDSA) cycles to close gaps.

4. **Outline seven key steps you will follow in managing these deaths as Patient Safety Incidents (PSIs) and explain how this supports clinical governance. (7 marks)**
  *Note to examiner: one mark per correctly named step with a brief explanation. Include notification, investigation, actioning and learning cycle*.
  - Step 1: Identify PSI and make safe: identify a maternal death as a PSI; preserve equipment and records; staff support.
  - Step 2: Immediate actions and disclosure: notify family and relevant authorities, such as the police forensic section (an autopsy is required for a maternal death); compassionate open disclosure; medico-legal processes for maternal death.
  - Step 3: Prioritisations: classify as SAC 1 (severity assessment code) PSI.
  - Step 4: Notification and reporting: notify management immediately; submit a notification to the provincial or district office within 24 h.
  - Step 5: Investigation: fill in the Patient Safety Incident Reporting Form during the 24-h maternal death review.
  - Step 6: Classification: clinical process error.
  - Step 7: Analysis: structured method (Root Cause Analysis [RCA], fishbone, London Protocol) integrating 24-h maternal death review; include human factors and environment.
  - Step 8: Implementation of recommendations, as per the root cause analysis during the maternal death review meeting: SMART actions with owners, deadlines and resources; monitoring indicators (e.g. capnography use, time-to-vasopressor, CS chart completeness).
  - Step 9: Learning: feedback to teams and family; update SOPs; schedule re-audit or PDSA; share at governance and district fora; document close-out.

### Further reading

- South Africa Department of Health. National integrated maternal and perinatal care guidelines for South Africa [homepage on the Internet]. 5th ed. Pretoria: South Africa Department of Health; 2024. Available from: https://knowledgehub.health.gov.za/system/files/elibdownloads/2024-10/Integrated%20Maternal%20and%20Perinatal%20Care%20Guideline_23_10_2024_0.pdf.
- National guideline for patient safety incident reporting and learning in South Africa (Version 2) [homepage on the Internet]. 2022 [2025 Nov 28]. Available from: https://knowledgehub.health.gov.za/system/files/elibdownloads/2022-03/National%20Guideline%20for%20Patient%20Safety%20Incident%20Reporting%20and%20Learning%20in%20South%20Africa%20Version%202_2022.pdf
- Kuün E, Spijkerman S. Curriculum development for the South African Essential Steps in Managing Obstetric Emergencies (ESMOE) anesthesiology training module: A Delphi study. Anaesth Analg. 2024;139(6):1181–1189. https://doi.org/10.1213/ANE.0000000000007019
- WHO surgical safety checklist [homepage on the Internet]. 2009 [cited 2025 Nov 25]. Available from: https://www.who.int/teams/integrated-health-services/patient-safety/research/safe-surgery/tool-and-resources
- Sotto KT, Burian BK, Brindle ME. Impact of the WHO surgical safety checklist relative to its design and intended use: A systematic review and meta-meta-analysis. J Am Coll Surg. 2021;233(6):794–809. https://doi.org/10.1016/j.jamcollsurg.2021.08.692
- Mazaza S, Gunst C. Chapter 8: Leadership and clinical governance. In Mash B, editor. Handbook of family medicine. 4th ed. Cape Town: Oxford University Press; 2017.

## Critical appraisal of research

Read the accompanying article carefully and answer the following questions. As far as possible, use your own words. Do not copy out chunks from the article. Be guided by the allocation of marks concerning the length of your responses.

Naidoo K, Spijkerman S, Wyngaard J, De Menezes-Williams H, Janse van Rensburg C. A cross-sectional observational study of endotracheal intubation and extubation practices among doctors treating adult COVID-19 and suspected COVID-19 patients in South Africa. S Afr Med J [serial online]. 2022 [cited 2025 Nov 28];112(1):40–48. Available from: http://www.samj.org.za/index.php/samj/article/view/13517

*This question was included in the Second Semester 2022 FCFP(SA) written paper*.

Total: 30 marks

### Questions

1. What are the key points in the argument for the social value of the study? (2 marks)
2. Comment on whether the authors’ research question was clearly focused in terms of the Population, Intervention or Issue of Interest, Comparator, Outcome (PICO) framework. (5 marks)
3. Comment critically on the sampling method used in this study and how the results reflect any selection bias. (6 marks)
4. Critically appraise how the authors ensured the validity of the study instrument and what role the expert panel played to ensure its validity. (5 marks)
5. Critically appraise the participation or response rate with reference to Figure 1 of the article under review. (2 marks)
6. Comment on the authors’ decision to use the Strengthening the Reporting of Observational Studies in Epidemiology (STROBE) guidelines? (2 marks)
7. Critically appraise the study finding that ‘intubations conducted in theatre environments had the lowest complication rate relative to other sites’. (2 marks)
8. Use the acronym READER (Relevance, Education, Applicability, Discrimination, Evaluation and Reaction) to analyse this article’s applicability to your own context (take-home message). (6 marks)

### Suggested answers

1. **What are the key points in the argument for the social value of the study? (2 marks)**
  Potential options include (*Two out of the three sentences [paraphrased] will be sufficient: the rationale for the research focuses on healthcare worker (HCW) safety, patient safety and adherence to guidelines to ensure the safety of the procedures*).
  - Data from the Severe Acute Respiratory Syndrome-related Coronavirus 1 (SARS-CoV-1) outbreak revealed that HCWs who performed endotracheal intubation of SARS-CoV-1-infected patients were 6.6 times more likely to contract SARS-CoV-1 compared with unexposed HCWs.
  - Furthermore, endotracheal intubation poses a considerable risk for complications in the physiologically compromised coronavirus disease 2019 (COVID-19) patient. Most COVID-19 patients requiring endotracheal intubation are likely to be tachypnoeic, tachycardic, hypotensive and of altered mentation, with 75.2% being hypoxaemic (peripheral pulse oximetry < 90%) before intubation.
  - Because of the risks to the patient and healthcare provider, various airway management guidelines have been developed to manage COVID-19 patients safely.
2. **Comment on whether the authors’ research question was clearly focused in terms of the PICO framework. (5 marks)**
  - P (patient group, patient problem or population of interest): COVID-19 patients requiring endotracheal intubation.
  - Intervention or Issue of Interest: the practice of endotracheal intubation and/or extubation of COVID-19 patients, especially regarding the use of or adherence to airway management guidelines in resource-poor healthcare settings.
  - Comparison intervention of interest: none of note, as the study design is observational or non-experimental.
  - Primary outcome of interest: mitigating the risks to the patient (considerable risk for complications exists in the physiologically compromised COVID-19 patient) and healthcare provider (these procedures are aerosol-generating and pose risks of SARS-CoV-2 exposure and transmission to the healthcare workers).
  - Therefore, yes, the research question was narrow and specific, as well as clearly focused on the PICO framework.
3. **Comment critically on the sampling method used in this study and how the results reflect any selection bias. (6 marks)**
  - The authors state (as the inclusion criteria) that all clinicians in South Africa performing endotracheal intubations and extubations in patients aged > 12 years with confirmed or suspected COVID-19 were considered potential study participants. No specific exclusion criteria were mentioned.
  - However, the questionnaire was distributed only to members of the South African Society of Anaesthesiologists (SASA) and shared with ‘representatives of other front-line disciplines for further distribution’. The ‘other front-line disciplines’ were not mentioned, and it is not clear what effort the authors made to ensure a representative pool of study participants, including the use of social media, WhatsApp or Telegram groups and email listservs. Table 2 confirms the selection bias towards SASA members and the discipline of anaesthesia, with 87% of respondents from the speciality of anaesthesiology, 5.1% from the related discipline of critical care, and less than 10% from other front-line disciplines.
  - The desired sample size was cited as 150 participants. The authors did not specify the statistical method or formula chosen to calculate the sample size, nor the effect size or reference used to determine the pre-study considerations of statistical power.
  - Furthermore, 59.4% of respondents were specialists, 30.4% were registrars and around 65% had more than 5 years’ experience (39.1% of this group had more than 10 years’ experience). This also shows a selection bias towards experienced providers with additional postgraduate training.
  - There is also a setting bias towards Gauteng, KwaZulu-Natal and the Western Cape provinces, which relates to higher levels of care, as these procedures occurred at higher levels of the health system.
  - There is a bias towards facilities with dedicated COVID-19 wards, Intensive Care Units (ICUs) and theatres, as reflected in the available difficult airway equipment (such as videolaryngoscopes), which precludes the typical district hospital environment.
4. **Critically appraise how the authors ensured the validity of the study instrument. (5 marks)**
  - *Content validity could be ensured using an expert panel*. In this study, the questionnaire was reviewed by several specialist anaesthesiologists affiliated with the University of Pretoria before distribution. *This panel may be deemed as representing sufficient expertise in the research topic*.
  - This expert panel would address the question of whether the content of the survey is related to the research aim and whether all relevant topics were included (and irrelevant topics excluded). Furthermore, the expert panel should determine whether the questions were formulated in a way that is likely to provide an in-depth and comprehensive exploration of the topic (i.e. *content validity*).
  - *Construct validity* involves using methods such as factor analysis to determine if the items included in the questionnaire measure the intended constructs. For example, do these items measure the construct of compassion? Construct validity was not considered by the expert panel in this study. An aspect of validity to consider, but may not have been relevant in this study.
  - While the authors describe the process of developing the questionnaire from local and international COVID-19 guidelines for endotracheal intubation and extubation practices, *they did not mention whether the expert panel review resulted in any changes or edits to the questionnaire*.
  - The authors also did not mention whether the questionnaire was *piloted* to confirm whether respondents found the questionnaire ‘brief and easy to administer’ and whether it took ‘3–7 minutes to complete’ as expected. *Piloting can address face validity* (do the intended respondents find the questionnaire design to be relevant and meaningful, and are the questions clear and logical?).
  - It is essential to describe the process undertaken to ensure the validity of the research instrument, including whether the questions elicit the desired response and whether they address the study’s aim and objectives. In the methods section, this description is incomplete. It does not guide the reader on the researchers’ efforts to prevent *information bias*, raising concern about possible flaws in the information-gathering process from study participants.
5. **Critically appraise the participation or response rate with reference to Figure 1 of the article under review. (2 marks)**
  - The authors stated that they were *unable to determine or calculate the exact participation rate* and cited the SASA membership base as a possible denominator. Still, they acknowledged that not all SASA members had managed COVID-19 patients and that this denominator excluded other disciplines that may have had access to the survey invitation.
  - Only 138 responses were analysed (135 intubations and 45 extubations, with both procedures performed on the same patient in some of the responses) after 36 of 174 responses had to be excluded because of lack of consent (4) or incomplete entry (32) – see Figure 1 in the article under review. The *sample size included in the analysis was therefore insufficient* compared to the target sample size of 150 participants.
6. **Comment on the authors’ use of the Strengthening the Reporting of Observational Studies in Epidemiology (STROBE) guidelines? (2 marks)**
  - The authors reported that ‘Data were reported in accordance with the Strengthening the Reporting of Observational Studies in Epidemiology (STROBE) guidelines’. The use of robust reporting guidelines, such as the STROBE guidelines, helps improve the reliability and value of published health research literature by promoting transparent and accurate reporting.
  - However, the authors did not comment on how this reporting guideline helped them identify any study limitations, as no further mention of the STROBE guideline appears in the manuscript other than the methods section. It would have been useful to include a brief statement on this at the end of the discussion section. Several limitations were found, but it was unclear whether the reporting guideline informed them.
7. **Critically appraise the study finding that ‘intubations conducted in theatre environments had the lowest complication rate relative to other sites’. (2 marks)**
  - The authors state that the theatre environment may result in intubations with lower complication rates, including the availability of equipment, drugs and skilled support staff, as well as potentially better team dynamics. However, the patients intubated in the theatre environment were likely more stable or less critically ill to allow for a more controlled, less urgent procedure execution, as the primary indication may have been for reasons other than respiratory failure, such as part of a surgical procedure.
  - This may reflect *information or measurement bias* if the dataset collation or subsequent analysis did not distinguish the complication rate relevant to the primary intubation indication and intubation setting. The authors acknowledged in the limitations section that the questionnaire did not capture indications for intubation, nor baseline characteristics and clinical parameters of patients who underwent intubation and extubation.
8. **Use the acronym READER (Relevance, Education, Applicability, Discrimination, Evaluation and Reaction) to analyse this article’s applicability to your own context (take-home message). (6 marks)**
  *The answer may be a subjective response, but it should reflect on possible changes in the student’s practice within the South African public healthcare system. It is acceptable for the student to suggest how his or her practice might change, within other scenarios after graduation (e.g. private general practice). The reflection on whether all essential outcomes were considered is, therefore, dependent on the reader’s own perspective (is there other information you would have liked to see?). A model answer could be written from the perspective of the family physician employed in the South African district health system:*
  - Relevance: This study is relevant to the African context, and there is a need to ensure safe intubations or extubations without compromising COVID-19 patient outcomes or increasing the risk of transmission to healthcare workers. However, the study team did not include primary care researchers, and very little of the data came from the primary care or district hospital settings, which reduces the study’s relevance. The selection bias in this study skews the findings towards an urban, tertiary, specialist-care environment. It would therefore not be possible to generalise the study findings to the broader South African district health services setting, where specialised equipment such as videolaryngoscopes, drugs such as cisatracurium infusions, and senior doctors experienced in difficult intubations are not readily available.
  - Education: The authors are motivated by increased adherence to guidelines and checklists that are feasible in low-resourced health settings. The preferred guidelines or checklists were not presented in this paper and only appeared as citations. A non-expert or non-specialist healthcare worker might have to search for these guidelines after reading the study’s findings. Potentially, these guidelines might point towards useful changes in practice, including challenging existing behaviour and beliefs.
  - Applicability: The typical Southern African district health services setting lacks access to some of the equipment and medications mentioned in the study. Some of the study’s findings and guidelines may be applied in the reader’s setting if the basic equipment cited is available and the scope of the procedures falls within the ambit of the district hospital setting.
  - Discrimination: In terms of discrimination, the internal validity of the study was compromised by the selection bias and information or measurement bias identified. The study did aim to address a clearly focused question, but the skewed sample and flaws in the data instrument compromised the usefulness of its findings.
  - Evaluation: The answer will reflect whether the study would change practitioner behaviour, clinical practice or service delivery. It is unlikely that this study will change policy direction, mainly because of its limitations, but it could help make the case for further research of a more robust design.
  - Reaction: The reaction would need to align with the evaluation above. The reaction would likely be that the research falls into the category of ‘this is of some interest’, but not enough to immediately change clinical practice, and further evidence should be sought. The study may be discussed with the local clinical team and used as a basis for reviewing the locally available resources and developing context-specific intubation and extubation guidelines.

### Further reading

- Von Elm E, Altman DG, Egger M, Pocock SJ, Gøtzsche PC, Vandenbroucke JP. The Strengthening the Reporting of Observational Studies in Epidemiology (STROBE) statement: Guidelines for reporting observational studies. Lancet. 2007;370(9596):1453–1457. https://doi.org/10.1016/S0140-6736(07)61602-X
- MacAuley D. READER: An acronym to aid critical reading by general practitioners. Br J Gen Pract. 1994;44(379):83.
- Von Pressentin KB, Shabani JS, Young T. Integrating evidence synthesis into doctoral research: A guide for family medicine and primary care. Afr J Prm Health Care Fam Med. 2025;17(2):a5198. https://doi.org/10.4102/PHCFM.v17i2.5198
- Von Pressentin KB, Motlhatlhedi K, Musende M, Schuster T. Real-world evidence for primary care: A primer on observational research. Afr J Prm Health Care Fam Med. 2025;17(2):a5197. https://doi.org/10.4102/PHCFM.v17i2.5197
- Buccheri RK, Sharifi C. Critical appraisal tools and reporting guidelines for evidence-based practice. Worldviews Evid Based Nurs. 2017;14(6):463–472. https://doi.org/10.1111/wvn.12258

## Objectively structured clinical examination station scenario

### Objective of the station

This station tests the candidate’s ability to safely plan and provide anaesthesia for an emergency caesarean section in a patient with co-existing cardiac disease (moderate mitral stenosis) and chronic hypertension in a resource-limited district hospital setting.

### Type of station

Integrated consultation

### Role player

A 30-year-old female in labour

### Instructions for the candidate

You are the family physician on call for anaesthesia at a district hospital. You are called urgently to the theatre to provide anaesthesia for an emergency caesarean section. The obstetrician has requested immediate surgery. The patient has a known history of chronic hypertension on methyldopa and moderate rheumatic mitral stenosis (MS) diagnosed three years ago, controlled with furosemide 40 mg daily. She last saw a cardiologist two years ago. She reports dyspnoea on exertion (New York Heart Association, NYHA II) but denies orthopnoea or paroxysmal nocturnal dyspnoea (PND). She is anxious, and she ate a light meal 2 h ago.

Your task:

- Conduct a focused pre-anaesthetic assessment and identify key maternal-foetal risks.
- Formulate and justify an appropriate anaesthetic plan.
- Explain your plan to the patient in a clear and reassuring manner.
- Demonstrate safe intra-operative and post-operative management strategies, including contingency plans.

### Instructions for the examiner

- This is an integrated consultation station assessing clinical reasoning, patient safety, leadership and communication in the provision of anaesthesia at the district level.
- The candidate has 20 min to complete this station.
- Familiarise yourself with the assessor guidelines, which detail the expected responses from the candidate.
- No marks are allocated. In the mark sheet, tick off one of the three responses for each competency listed in Table 1. Ensure you are clear on the criteria for judging a candidate’s competence in each area.

**TABLE 1:** Marking sheet for consultation station.

Competencies	Candidate’s rating		
	Not competent	Competent	Good
Establishes and maintains a good doctor-patient relationship			
Gathering information: history/examination/investigations			
Clinical reasoning			
Explanation and planning			
Management			

### Guidance for examiners

The station assesses whether the candidate can:

- Demonstrate a structured pre-anaesthetic assessment under emergency conditions.
- Identify anaesthetic risks in a pregnant patient with mitral stenosis and hypertension.
- Provide safe, evidence-based obstetric anaesthesia using available resources.
- Anticipate complications (pulmonary oedema, hypotension, arrhythmia, failed spinal, airway issues and preparedness for general anaesthesia).
- Outline post-operative monitoring, analgesia and handover.
- Apply district-hospital resource awareness (limited ICU, blood products, monitoring).

A working definition of competent performance: the candidate effectively completes the task within the allotted time, in a manner that maintains patient safety, even though the execution may not be efficient and well-structured:

- Not competent: patient safety is compromised (including ethically and legally), or the task is not completed.
- Competent: the task is completed safely and effectively.
- Good: in addition to demonstrating competence, the task is completed efficiently and in an empathetic, patient-centred manner (acknowledging the patient’s ideas, beliefs, expectations, concerns or fears).

1. **Establishes and maintains a good clinician–patient relationship:**
  The competent candidate introduces themselves, confirms the patient’s identity and explains their role. Maintains composure, shows respect and empathy. Reassures patient about urgency and safety: ‘We will take good care of you and your baby’. Maintains privacy and cultural sensitivity. In addition to the above, the good candidate builds a strong rapport under stress through a calm, confident tone and active listening. Validates fears (‘It is normal to be anxious when things move quickly’). Adapts language to the patient’s level, avoids jargon. Demonstrates leadership by creating a sense of calm in the theatre team and models empathy to staff.
2. **Gathering information**
  The competent candidate elicits full medical and obstetric history: rheumatic heart disease, degree of MS, medications, NYHA class, previous anaesthetics and allergies. Screens for red flags: orthopnoea, PND, palpitations, chest pain, cough with froth. Performs targeted examination: pulse rhythm, BP, Jugular Venous Pressure (JVP), auscultation (mid-diastolic murmur, loud S1), signs of heart failure, airway, Mallampati, dentition. Reviews vitals (BP 160/100, HR 92 bpm, RR 22/min, SpO_2_ 98 %), Full Blood Count (FBC), platelets, ECG (sinus rhythm), available echo summary (moderate MS, mean gradient 6 mmHg). The examiner provides this upon request. In addition to the above, the good candidate integrates biopsychosocial and contextual assessment: theatre readiness, skilled assistant availability, suction, oxygen supply, vasopressors, blood and postoperative bed. Anticipates need for multi-disciplinary coordination (obstetrician, nurse, neonatal team). Actively identifies resource constraints and develops mitigation strategies (e.g. judiciously preload fluids because of MS, plan for early referral if complications).
3. **Clinical reasoning**
  The competent candidate recognises dual pathology: obstetric emergency and cardiac disease. Correctly classifies urgency (Category 1 – foetal distress). Identifies key risks: pulmonary oedema, tachycardia, hypotension, aspiration. Chooses graded or single-shot spinal anaesthesia with low-dose local anaesthetic and opioid *if haemodynamically stable and time allows*; or ketamine-assisted GA with invasive airway management *if urgent and contraindications exist*. Plans for controlled preload, avoidance of fluid overload, readiness to manage hypotension with phenylephrine (preferred) or ephedrine cautiously.

In addition to the above, the good candidate justifies the technique in detail:

- Explains that MS patients poorly tolerate tachycardia and sudden preload or afterload changes.
- Avoids agents that increase HR (ketamine high dose, atropine).
- Prepares for GA only with full RSI precautions and cardiac monitoring if spinal not feasible. Anticipates obstetric complications (postpartum haemorrhage) and coordinates early blood availability. Prioritises maternal safety while balancing foetal urgency; demonstrates sound ethical reasoning and systems awareness.

4. **Explaining and planning**
  The competent candidate uses simple, clear language to describe anaesthetic plan, risks, monitoring and postoperative care. Obtains consent verbally. Explains need for emergency surgery: ‘Your baby’s heartbeat shows distress, and we must deliver now’. Outlines anaesthetic plan simply: ‘You will get an injection in your back to make you numb from the chest down; you will stay awake but will not feel pain’. Reassures about monitoring, staff presence and postoperative care. In addition to the above, the good candidate provides a comprehensive, structured explanation using calm pacing and empathy. Addresses cardiac risk in lay terms: ‘Your heart valve is a little narrow, so we will work carefully to keep your heartbeat steady and your breathing comfortable’. Checks understanding, invites questions and ensures informed consent (even under emergency conditions). Demonstrates leadership and communication skills – informs the surgeon and nurse of the anaesthetic plan and confirms the readiness checklist aloud.
5. **Management**
  The competent candidate will state:
  **Pre-operative:**
  - Pre-oxygenate, secure large-bore IV, and connect full monitoring (ECG, NIBP, pulse ox).
  - Antacid prophylaxis (30 mL sodium citrate ± ranitidine).
  - Minimal preload (200 mL – 300 mL crystalloid), avoid fluid overload.
  - Prepare phenylephrine, ephedrine, furosemide, airway rescue equipment and suction.
  **Intra-operative (if spinal):**
  - Low-dose hyperbaric bupivacaine + fentanyl (e.g. 1.6 mL – 1.8 mL).
  - Left lateral tilt, avoid rapid position changes.
  - Maintain HR 60 bpm – 80 bpm, treat hypotension with phenylephrine.
  **Intra-operative (if GA):**
  - RSI with cricoid pressure, avoid tachycardia (use fentanyl 1 µg/kg – 2 µg/kg, low-dose induction).
  - Maintain oxygenation, normocapnia and gentle ventilation to prevent pulmonary congestion.
  **Post-operative:**
  - Observe in high-care or monitored recovery for ≥ 24 h.
  - Continue furosemide, monitor fluid balance and urine output.
  - Use of multimodal analgesia (paracetamol, regional block and local infiltration). Avoid excessive fluids.

In addition to the above, the good candidate leads a full WHO Surgical Safety Checklist, confirms emergency drugs, blood and neonatal resuscitation readiness. Plans for arrhythmia or pulmonary oedema management (oxygen, furosemide, morphine, cautious fluids). Ensures handover and continuity: detailed anaesthetic note, cardiac follow-up referral, postpartum cardiology review. Reflects on district-level limitations and articulates when referral or telephonic consultation with a regional anaesthetist would be warranted.

### Role play: Instructions for the actor (Table 2)

**Name:** Mrs Mkhize

**Age:** 30 years

**Gravida/Para:** G2 P1

**Gestation:** 39 weeks

**TABLE 2:** Examination findings and investigation.

Parameter	Finding
BP	160/100 mmHg
HR	92 bpm, regular
RR	22/min
SpO_2_	98 % RA
JVP	6 cm of water
Apex	Tapping
Murmur	Mid-diastolic @ apex, loud S1
CXR	Mild left atrial enlargement
ECG	Sinus rhythm, left atrial enlargement
Echo summary	Moderate MS, MVA 1.5 cm^2^, mean gradient 6 mmHg, PA pressure 35 mmHg
Hb	11 g/dL
Platelets	210 × 10⁹/L

**Past medical history:** Chronic hypertension on Methyldopa 250 mg twice daily, moderate rheumatic MS diagnosed three years ago after an episode of shortness of breath during her previous pregnancy, on Furosemide 40 mg once daily. Last cardiology visit two years ago (no recent echocardiogram). No known allergies.

**Functional status:** Gets breathless when walking up a hill or climbing more than one flight of stairs (NYHA class II). Sleeps with two pillows; denies waking up gasping (no orthopnoea or PND). No palpitations or chest pain experienced.

**Obstetric history:** previous vaginal delivery at term, no complications. The current pregnancy was uneventful until now. Ate a sandwich and juice 2 h ago; Foetal distress confirmed; on-call obstetrician recommends immediate delivery.

### Opening line (to start the consultation)

‘Doctor, the nurses said my baby’s heart is in trouble … is it safe for me to have this operation with my heart problem?’

You are very frightened and uncertain. You want reassurance that you, and your baby, will survive the operation.

### Further reading

- Van Rensburg G, Van Dyk D, Bishop D, et al. The management of high spinal anaesthesia in obstetrics: Suggested clinical guideline in the South African context. S Afr J Anaesth Analg. 2016;22(suppl 1):S1–S5.
- South African Society of Anaesthesiologists (SASA). Practice guidelines – 2022 revision. S Afr J Anaesth Analg. 2022;28(4):161–163. https://doi.org/10.36303/SAJAA.2022.28.4.S1.2851

